# ANNOUNCEMENT / NOUVELLE

**DOI:** 10.29173/jchla29940

**Published:** 2026-04-01

**Authors:** Marie-Hélène Nicol

**Affiliations:** Chair, CHLA/ABSC 2026 Conference

*[Le français suit]*

**Join us in Québec City from June 2 to 5, 2026 for CHLA/ABSC’s 50th Anniversary Conference!**

We are delighted to invite you to Québec City, Québec, for the 2026 Annual Conference of the Canadian Health Libraries Association / Association des bibliothèques de la santé du Canada (CHLA/ABSC), taking place from June 2–5, 2026. This milestone year marks 50 years of the Association, celebrated under the theme “Honouring the Past, Shaping the Future: 50 Years of Health Expertise.”

Our conference venue, Hotel Le Concorde, offers an excellent setting for reflecting on our history while looking ahead to the future of health information practice.

The 2026 conference will welcome colleagues from across Canada and beyond for a full program featuring contributed papers, lightning talks, workshops, panels, and posters, along with special anniversary activities recognizing five decades of professional accomplishments.

We are honoured to welcome two distinguished invited speakers:

Isabelle Picard—Wednesday, June 3rd, 2026. An ethnologist, author, speaker, and columnist specializing in First Nations realities, Isabelle Picard brings extensive experience in cultural revitalization and public scholarship.

Marie-Pierre Gagnon (Université Laval)—Friday, June 5th, 2026. A leading voice in digital health research and Canada Research Chair in Health Technologies and Practices.

A commemorative discussion on the past, present, and future of CHLA/ABSC and of health librarianship will take place during the 50th Anniversary Panel—Thursday, June 4th, 2026. Moderator Lee-Anne Bourke, Associate Librarian at the University of Ottawa and CHLA/ABSC Past President (2014–2015), will host panelists David S. Crawford (CHLA/ABSC founder and First President), Kristy Hancock (2023 Emerging Leader Award, UNBC), Martine Gagnon (incoming CHLA/ABSC President, Université Laval), and Janice Thompson (Manager, Library and Research Services, William Osler Health System, Brampton Civic Hospital).

Registration will end on April 24th, 2026. As in previous years, members of the Medical Library Association (MLA) are eligible for CHLA/ABSC member rates.

We will officially launch the conference on Tuesday, June 2nd, 2026, with a social activity and the Welcome Reception, providing a warm opportunity to reconnect with colleagues and begin our 50th anniversary celebrations.

On behalf of the 2026 Planning Committee and the FMD3S Québec Chapter, I look forward to welcoming you to Québec City as we honour our past and shape our future together.

**Marie-Hélène Nicol**

*Chair, CHLA/ABSC 2026 Conference*

For program updates and conference details, please visit:

https://www.chla-absc.ca/welcome_to_chla_2026.php

**Joignez-vous à nous à Québec du 2 au 5 juin 2026 pour célébrer le 50e anniversaire de l’ABSC/CHLA !**

Nous sommes ravi•e•s de vous inviter dans la ville de Québec, au Québec, pour le Congrès annuel 2026 de l’Association des bibliothèques de la santé du Canada / Canadian Health Libraries Association (ABSC/CHLA), qui se tiendra du 2 au 5 juin 2026. Cette édition marquante souligne le 50^e^ anniversaire de l’Association, célébré sous le thème: « Honorer la mémoire, façonner l’avenir : 50 ans d’expertise en santé ».

Le lieu choisi pour le congrès, l’Hôtel Le Concorde, offre un cadre idéal pour réfléchir à notre histoire tout en portant un regard vers l’avenir de nos pratiques professionnelles.

Le congrès 2026 accueillera des collègues de partout au Canada et d’ailleurs pour un programme complet comprenant des communications, des communications éclair, des ateliers, des panels et des affiches scientifiques, ainsi que des activités spéciales soulignant cinq décennies d’accomplissements professionnels.

Nous avons l’honneur d’accueillir deux conférencières invitées de renom :

Isabelle Picard—mercredi 3 juin 2026. Ethnologue, autrice, conférencière et chroniqueuse spécialisée dans les réalités des Premières Nations, Isabelle Picard possède une vaste expertise en revitalisation culturelle et en diffusion des savoirs.

Marie-Pierre Gagnon (Université Laval)—vendredi 5 juin 2026. Une figure de proue de la recherche en santé numérique et titulaire de la Chaire de recherche du Canada en technologies et pratiques de santé.

Une discussion commémorative sur le passé, le présent et l’avenir de l’ABSC/CHLA et de la bibliothéconomie en santé aura lieu dans le cadre du panel du 50^e^ anniversaire—jeudi 4 juin 2026. Lee Anne Bourke, bibliothécaire associée à l’Université d’Ottawa et ancienne présidente de l’ABSC/CHLA (2014–2015), animera un panel composé de David S. Crawford (fondateur et premier président de l’ABSC/CHLA), Kristy Hancock (lauréate 2023 du prix du flambeau de la relève, UNBC), Martine Gagnon (présidente désignée de l’ABSC/CHLA, Université Laval) et Janice Thompson (gestionnaire, Services de bibliothèque et de recherche, William Osler Health System, Brampton Civic Hospital).

Les inscriptions prendront fin le 24 avril 2026. Comme les années précédentes, les membres de la Medical Library Association (MLA) sont admissibles au tarif membre de l’ABSC/CHLA.

Le congrès sera officiellement lancé le mardi 2 juin 2026 à l’occasion d’une activité sociale et une réception de bienvenue, offrant un moment privilégié pour renouer avec les collègues et amorcer ensemble les célébrations de notre 50^e^ anniversaire.

Au nom du comité organisateur 2026 et de la FMD3S, chapitre québécois, j’ai hâte de vous accueillir à Québec alors que nous honorerons notre passé et façonnerons ensemble notre avenir.

**Marie-Hélène Nicol**

*Présidente, Congrès ABSC/CHLA 2026*

Pour les mises à jour du programme et les détails du congrès, veuillez visiter:

https://www.chla-absc.ca/bienvenue_a_absc_2026.php

